# Supplementary material for: The release of inhibition model reproduces kinetics and plasticity of neurotransmitter release in central synapses
Source: Commun Biol. 2023 Oct 27;6:1091. doi: 10.1038/s42003-023-05445-2 (PMC10611806; doi:10.1038/s42003-023-05445-2)
Supplement: Supplementary file 2 — Supplementary Information [file 42003_2023_5445_MOESM2_ESM.pdf]

Supplementary Information

Title: The release of inhibition model reproduces kinetics and plasticity of neurotransmitter release in central synapses.

Christopher A Norman[1,2,3], Shyam S Krishnakumar[1,4,*], Yulia Timofeeva[1,2,*] and Kirill E Volynski[1,5,*].

[1]University College London Institute of Neurology, University College London, London, WC1N 3BG, UK;

[2]Department of Computer Science, University of Warwick, Coventry, CV4 7AL, UK;

[3]Mathematics for Real-World Systems Centre for Doctoral Training, University of Warwick, Coventry, CV4 7AL, UK;

[4]Department of Neurology, Yale Nanobiology Institute, Yale University School of Medicine, New Haven, CT 06510, USA;

[5]Department of Cell Biology, Yale University School of Medicine, New Haven, CT 06510, USA;

*For correspondence:

k.volynski@ucl.ac.uk

y.timofeeva@warwick.ac.uk

shyam.krishnakumar@yale.edu

This PDF file includes:

Supplementary Figures 1 to 5

Supplementary Tables 1 and 2

Supplementary Note 1

Supplementary References

Other Supplementary Materials for this manuscript include the following:

Norman_et_al_code.zip, custom MATLAB codes used in this work

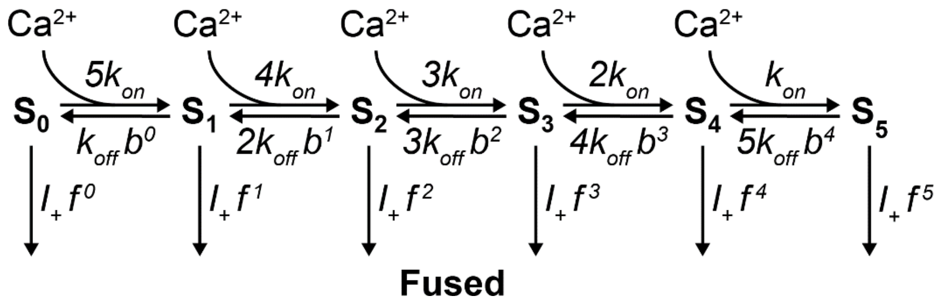

Supplementary Figure 1. Benchmark allosteric model

Six-state allosteric model of Ca^{2+} activation of vesicle fusion in the calyx of Held described in ref. [1]. The model parameters are: k_{on} = 0.1 μM^{-1} ms^{-1}, k_{off} = 4 ms^{-1}, b = 0.5, f = 31.3, and I_+ = 2 × 10^{-7} ms^{-1}. The model was stochastically simulated identically to the release of inhibition models (see Methods).

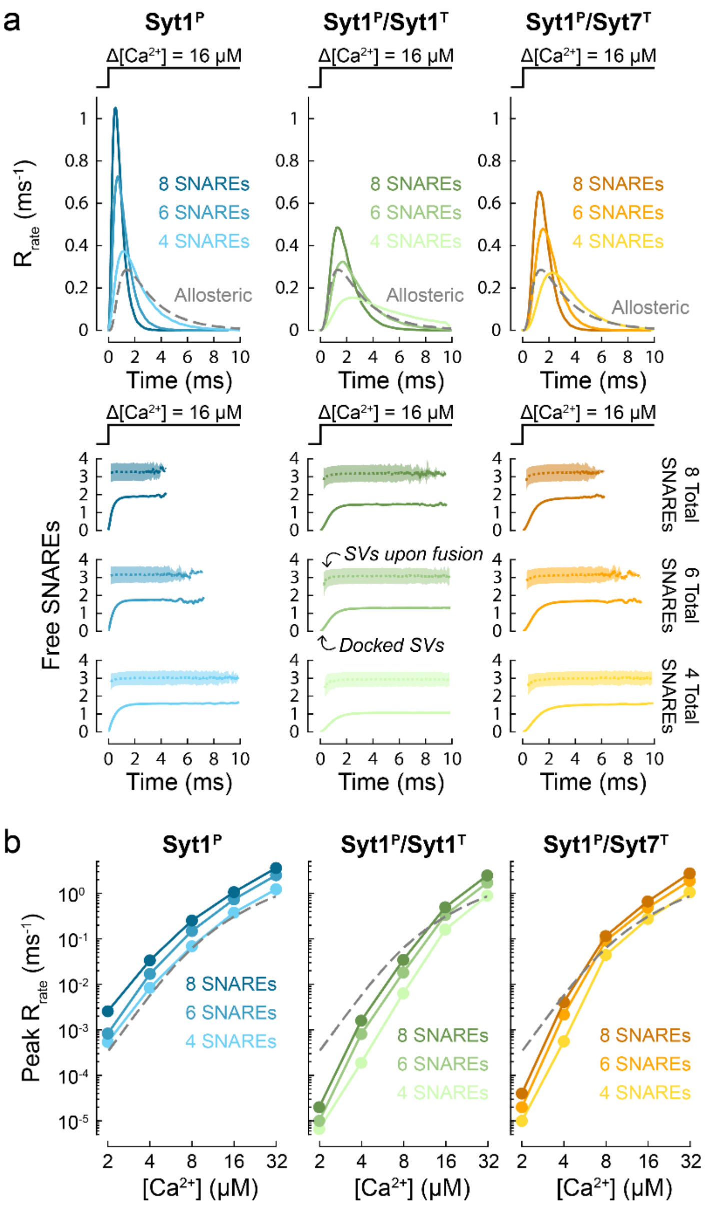

Supplementary Figure 2. Effect of total number of SNAREpins on Ca2+ activation of SV fusion.

a) Top, time-course of vesicular release rate simulated in response to a 16 μM $[Ca^{2+}]$ step for the single and dual synaptotagmin/SNARE clamp architectures considered in the model with four, six, or eight total SNAREpins per vesicle (solid coloured traces) and for the benchmark allosteric model (dashed grey trace). Bottom, time evolution of the mean number of unclamped SNAREpins ('Free SNAREs') on all docked SVs (solid lines), and on SVs at the instance of fusion (dotted lines) in response to the 16 μM $[Ca^{2+}]$ step. Shaded area indicates 1 standard deviation each side of the mean. Each time point includes data from a 0.15 ms bin.

b) Dependency of the peak release rate (achieved within 10 ms) on the amplitude of the $[Ca^{2+}]$ step. For each $[Ca^{2+}]$ step and fusion clamp architecture at least *N* = 100,000 stochastic simulations were performed with at least 1,000 vesicular fusion events recorded during the first 10 ms time window.

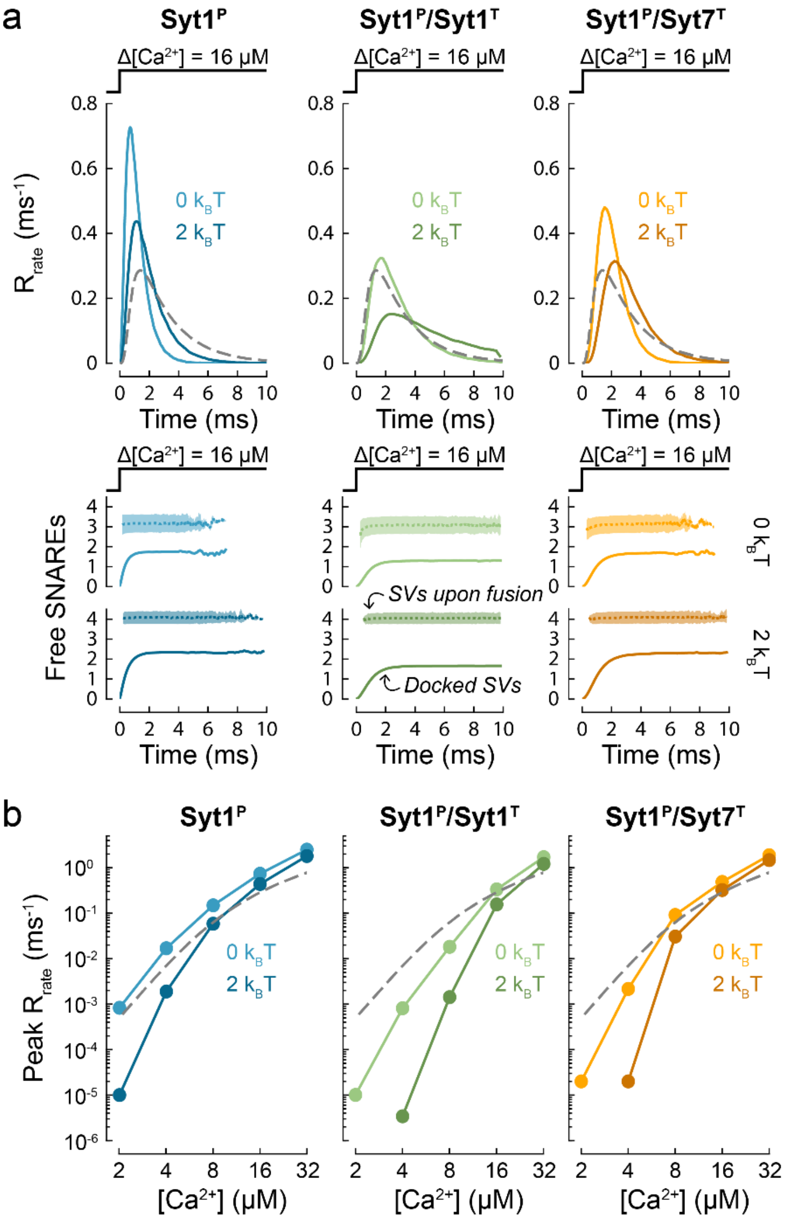

Supplementary Figure 3. Effect of mechanical coupling among SNAREpins on the same vesicle on Ca2+ activation of SV fusion.

To model mechanical coupling, we assumed that each clamped SNAREpin acts as an additional mechanical obstacle for the fusing membranes, thereby generating a negative feedback loop by introducing an extra energy barrier of 2 k_BT.

a) Top, time-course of vesicular release rate simulated in response to a 16 μM $[Ca^{2+}]$ step for the single and dual synaptotagmin/SNARE clamp architectures considered, with each unclamped SNAREpin contributing either 0 k_BT or 2 k_BT to the total energy barrier (solid coloured traces) and for the benchmark allosteric model (dashed grey trace). Bottom, time evolution of the mean number of unclamped SNAREpins ('Free SNAREs') on all docked SVs (solid lines), and on SVs at the instance of fusion (dotted lines) in response to the 16 μM $[Ca^{2+}]$ step. Shaded area indicates 1 standard deviation each side of the mean. Each time point includes data from a 0.15 ms bin.

b) Dependency of the peak release rate (achieved within 10 ms) on the amplitude of the $[Ca^{2+}]$ step. For each $[Ca^{2+}]$ step and fusion clamp architecture at least N = 100,000 stochastic simulations were performed with at least 1,000 vesicular fusion events recorded during the first 10 ms time window. For clarity, only points with release rate > 10^{-6} ms^{-1} are shown.

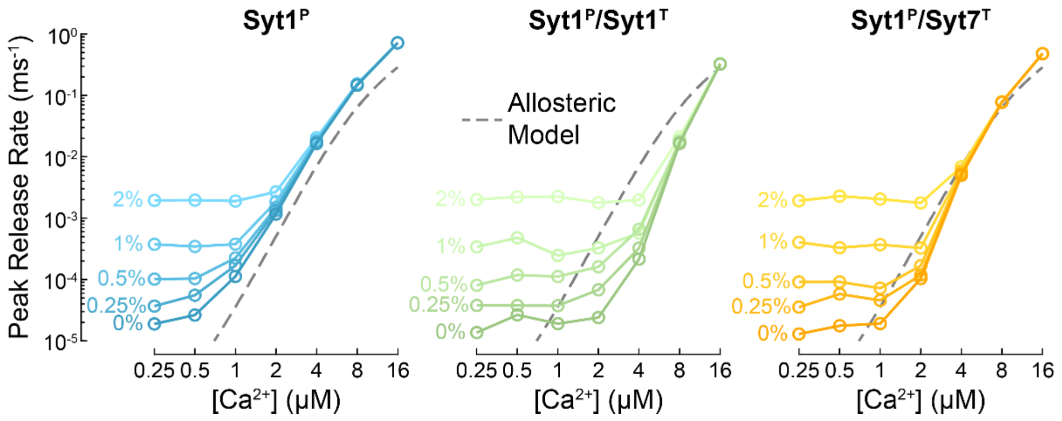

Supplementary Figure 4. Modelling of spontaneous release of fusion clamp

Dependency of the peak release rate (achieved within 20 ms) on the amplitude of the $[Ca^{2+}]$ step for a range of cases in which the probability that each individual SNAREpin was initially unclamped varied between 0 - 2%, as indicated. For each $[Ca^{2+}]$ step at least *N* = 200,000 stochastic simulations were performed with at least 2,000 vesicular fusion events recorded.

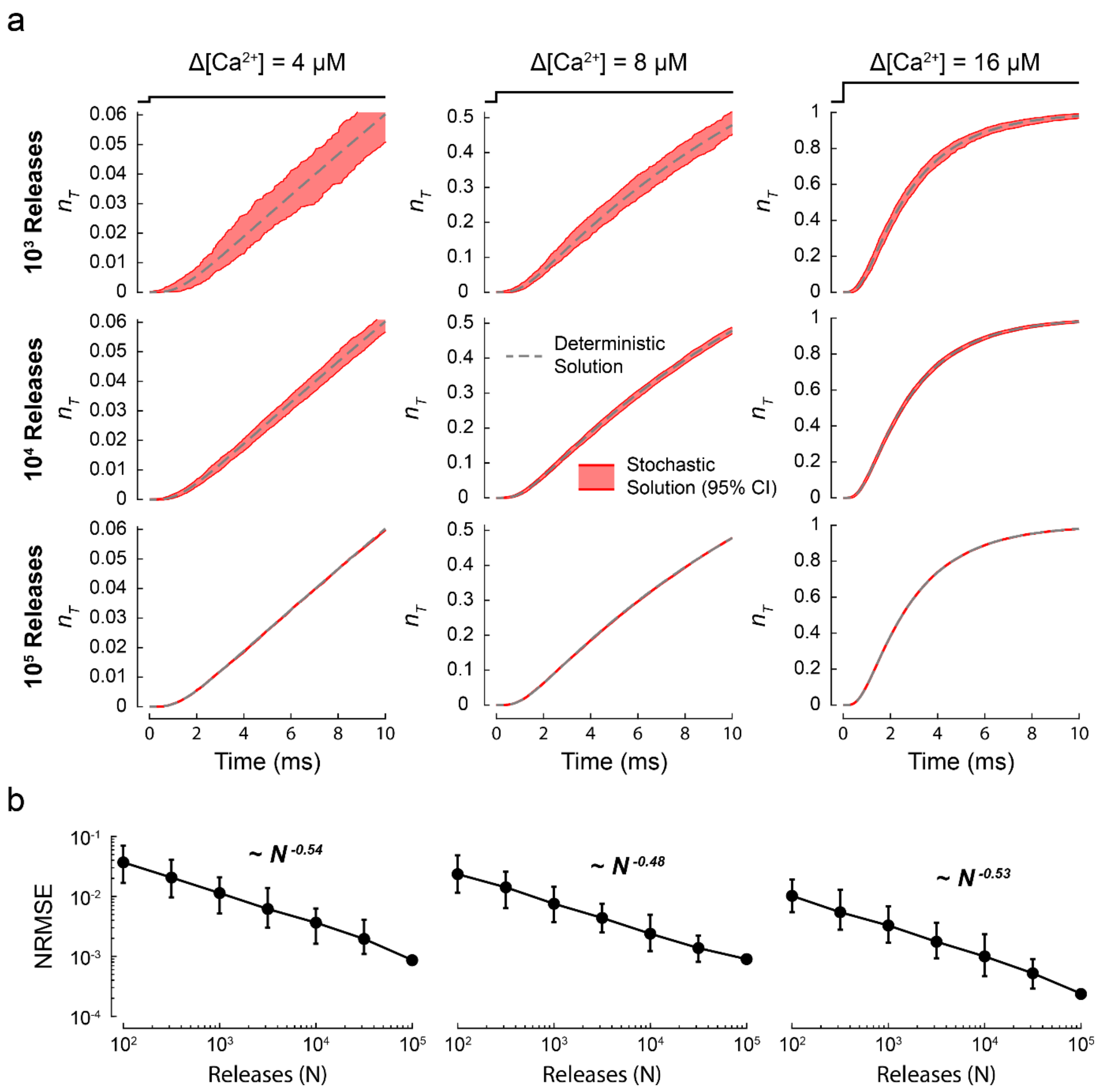

Supplementary Figure 5. Convergence of Monte Carlo estimates to exact solution with the allosteric model.

Stochastic simulations of the allosteric model were performed in response to 100 ms $[Ca^{2+}]$ steps at 4, 8, and 16 μM, without vesicle replenishment, until 100,000 release events were achieved. Monte Carlo estimates of $n_T(t)$ were then calculated, as described in Methods, from subsamples drawn from these release event collections and compared against the exact solutions of the differential master equations.

a) The first 10 ms of simulations in response to each $[Ca^{2+}]$ step (indicated above) for subsample sizes of 1,000, 10,000, and 100,000 release events with 95% confidence intervals calculated from 100 subsamples (shaded areas).

b) The typical deviation of Monte Carlo estimates with N stochastic simulations from the deterministic solution was quantified as the normalised root mean squared error (NRMSE) which is given by

$$NRMSE = \frac{\sqrt{\frac{1}{N}\sum_{i=1}^{N}\left(Y_i - \hat{Y}_i\right)^2}}{\max_i\left(\hat{Y}_i\right) - \min_i\left(\hat{Y}_i\right)}$$

where Y_i are the Monte Carlo predictions and $\hat{Y}_i$ are the corresponding deterministic values. The NRMSE is shown for release time subsample sizes between 100 and 100,000. Data are presented as mean values with 95% confidence intervals calculated from 100 subsamples. Slopes of best fit over the range are shown, indicating that NRMSE scales with the number of recorded release times, N, roughly according to $\frac{1}{\sqrt{N}}$. When all 100,000 release times are used to generate Monte Carlo predictions the maximal NRMSE is 0.0867%. In the worst-case scenario where only 2,250 release times are recorded, the maximal NRMSE (estimated from linear interpolation between mean values) is 0.898%.

Supplementary Table 1. Ca^{2+} and membrane binding properties of Syt1 and Syt7 used in the model.

Parameter	Syt1	Syt7
k_{on}	1 μM^{-1} ms^{-1}	1 μM^{-1} ms^{-1}
k_{off}	150 ms^{-1}	150 ms^{-1}
k_{in}	100 ms^{-1}	100 ms^{-1}
k_{out}	0.67 ms^{-1}	0.02 ms^{-1}

Supplementary Table 2. Properties of Ca^{2+} buffers used in VCell models of presynaptic Ca^{2+} dynamics.

Ca^{2+} (refs.[2, 3])

$D_{Ca^{2+}}$	0.22 μm^2 ms^{-1}

Calbindin-D$_{28K}$ (refs.[3-6])

Reactions:

$$CB_{fast} + Ca^{2+} \underset{k_{off}^{CB\_fast}}{\overset{k_{on}^{CB\_fast}}{\rightleftarrows}} CaCB_{fast}$$

$$CB_{slow} + Ca^{2+} \underset{k_{off}^{CB\_slow}}{\overset{k_{on}^{CB\_slow}}{\rightleftarrows}} CaCB_{slow}$$

Reaction rates:

$k_{on}^{CB\_fast}$	8.7 x 10^{-2} μM^{-1} ms^{-1}
$k_{off}^{CB\_fast}$	3.58 x 10^{-2} ms^{-1}
$[CB_{fast}]_{total}$	95 μM
$k_{on}^{CB\_slow}$	1.1 x 10^{-2} μM^{-1} ms^{-1}
$k_{off}^{CB\_slow}$	2.6 x 10^{-2} ms^{-1}
$[CB_{slow}]_{total}$	95 μM
D_{CB}	2 x 10^{-2} μm^2 ms^{-1}

ATP (refs.[2, 3, 7])

Reactions:

$$ATP + Ca^{2+} \underset{k_{off}^{ATP}}{\overset{k_{on}^{ATP}}{\rightleftarrows}} CaATP$$

Reaction rates:

k_{on}^{ATP}	0.5 μM^{-1} ms^{-1}
k_{off}^{ATP}	100 ms^{-1}
$[ATP]_{total}$	0.9 mM (corresponding to 58 μM $[ATP]_{free}$ at 1 mM $[Mg^{2+}]_{free}$ [7])
D_{ATP}	0.22 μm^2 ms^{-1}

Calmodulin (Supplementary refs.[7, 8])

Reactions:

N-lobe

$$N_T N_T + Ca^{2+} \underset{k_{off}^{(T),N}}{\overset{2\cdot k_{on}^{(T),N}}{\rightleftarrows}} CaN_T N_R + Ca^{2+} \underset{2\cdot k_{off}^{(R),N}}{\overset{k_{on}^{(R),N}}{\rightleftarrows}} CaN_R CaN_R$$

C-lobe

$$C_T C_T + Ca^{2+} \underset{k_{off}^{(T),C}}{\overset{2\cdot k_{on}^{(T),C}}{\rightleftarrows}} CaC_T C_R + Ca^{2+} \underset{2\cdot k_{off}^{(R),C}}{\overset{k_{on}^{(R),C}}{\rightleftarrows}} CaC_R CaC_R$$

Reaction rates:

N-lobe

$k_{on}^{(T),N}$	0.77 μM^{-1} ms^{-1}
$k_{off}^{(T),N}$	160 ms^{-1}
$k_{on}^{(R),N}$	32 μM^{-1} ms^{-1}

$k_{off}^{(R),N}$	22 ms^{-1}
<u>C-lobe</u>	
$k_{on}^{(T),C}$	8.4 x 10^{-2} μM^{-1} ms^{-1}
$k_{off}^{(T),C}$	2.6 ms^{-1}
$k_{on}^{(R),C}$	2.5 x 10^{-2} μM^{-1} ms^{-1}
$k_{off}^{(R),C}$	6.5 x 10^{-3} μM^{-1} ms^{-1}
$[CaM]_{total}$	100 μM
D_{CaM}	2 x 10^{-2} μm^2 ms^{-1}

Supplementary Note 1. Estimation of Syt1 and Syt7 C2 domain membrane dissociation rates from stopped-flow experimental data.

The dissociation kinetics of Syt1 and Syt7 C2 domains from the membranes have previously been determined in stopped flow experiments where Ca^{2+} is rapidly removed from the system after fast dilution in an EGTA-containing buffer. The membrane dissociation curves closely follow single exponential decay functions with dissociation rate constants (k_{diss}) in the range of 0.38 – 0.7 ms^{-1} for Syt1 and 0.008 – 0.02 ms^{-1} for Syt7 [9-11]. In this work we used representative values of k_{diss} = 0.5 ms^{-1} for Syt1 and k_{diss} = 0.015 ms^{-1} for Syt7.

Modelling the stopped flow experiment, the kinetic scheme of Ca^{2+} binding and membrane insertion of synaptotagmin C2 domains shown in Figure 1B can be reduced to $S_{0-1} \xleftarrow{2k_{off}} S_2 \underset{k_{out}}{\overset{k_{in}}{\rightleftarrows}} I$. Indeed, when Ca^{2+} is removed from the system the lowest states in the kinetic model in Figure 1B (states S_0 and S_1), can be combined into a single absorbing state S_{0-1}. The dynamics of the non-absorbing states, S_2 and I, are described by the system of first order ordinary differential equations:

$$\frac{dP(S_2)}{dt} = k_{out}P(I) - (k_{in} + 2k_{off})P(S_2),$$

$$\frac{dP(I)}{dt} = k_{in}P(S_2) - k_{out}P(I),$$

where $P(A)$ indicates the probability that the system occupies state A at time t. The general solution for this system can be expressed as $P(I) = Ae^{\lambda_1 t} + Be^{\lambda_2 t}$, where A and B are constants and the rate parameters are given by

$$\lambda_1 = \frac{-(k_{out} + 2k_{off} + k_{in}) + \sqrt{(k_{out} + 2k_{off} + k_{in})^2 - 8k_{off}k_{out}}}{2},$$

$$\lambda_2 = \frac{-(k_{out} + 2k_{off} + k_{in}) - \sqrt{(k_{out} + 2k_{off} + k_{in})^2 - 8k_{off}k_{out}}}{2}.$$

From the solutions for λ_1 and λ_2, using the constrained values of $k_{on} = 1$ μM^{-1} ms^{-1}, k_{off} = 150 ms^{-1}, and k_{in} = 100 ms^{-1}, the fast component λ_2 has a magnitude greater than 400 ms^{-1} for all positive values of k_{out}, and would therefore dissipate well within the dead time of the stopped-flow apparatus (> 1 ms) [10]. This means that the slower exponential component dominates the model dynamics over timescales observed in the stopped-flow experiments, and the simplification $P(I) \propto e^{\lambda_1 t}$ should provide an appropriate approximation of the experimental data. The full expansion of λ_1 can then be equated with the apparent membrane

dissociation rate of Syt1 or Syt7, k_{diss}, to complete the system of kinetic parameters with: $k_{out} = k_{diss}\left(1 - \frac{k_{in}}{k_{diss} - 2k_{off}}\right)$. For the value of k_{diss} = 0.5 ms^{-1} this equation yields k_{out} = 0.67 ms^{-1} for Syt1, and for k_{diss} = 0.015 ms^{-1} it yields k_{out} = 0.02 ms^{-1} for Syt7.

Supplementary References

1. Lou,X., Scheuss,V., & Schneggenburger,R. Allosteric modulation of the presynaptic Ca2+ sensor for vesicle fusion. Nature. 435, 497-501 (2005).

2. Meinrenken,C.J., Borst,J.G., & Sakmann,B. Calcium secretion coupling at calyx of held governed by nonuniform channel-vesicle topography. J. Neurosci. 22, 1648-1667 (2002).

3. Goswami,S.P., Bucurenciu,I., & Jonas,P. Miniature IPSCs in Hippocampal Granule Cells Are Triggered by Voltage-Gated Ca2+ Channels via Microdomain Coupling. J. Neurosci. 32, 14294-14304 (2012).

4. Eggermann,E., Bucurenciu,I., Goswami,S.P., & Jonas,P. Nanodomain coupling between Ca(2+) channels and sensors of exocytosis at fast mammalian synapses. Nat. Rev. Neurosci. 13, 7-21 (2011).

5. Naraghi,M. T-jump study of calcium binding kinetics of calcium chelators. Cell Calcium. 22, 255-268 (1997).

6. Nagerl,U.V., Novo,D., Mody,I., & Vergara,J.L. Binding kinetics of calbindin-D(28k) determined by flash photolysis of caged Ca(2+). Biophys. J. 79, 3009-3018 (2000).

7. Faas,G.C., Raghavachari,S., Lisman,J.E., & Mody,I. Calmodulin as a direct detector of Ca2+ signals. Nat. Neurosci. 14, 301-304 (2011).

8. Xia,Z. & Storm,D.R. The role of calmodulin as a signal integrator for synaptic plasticity. Nat. Rev. Neurosci. 6, 267-276 (2005).

9. Hui,E. et al. Three distinct kinetic groupings of the synaptotagmin family: candidate sensors for rapid and delayed exocytosis. Proc. Natl. Acad. Sci. U. S. A. 102, 5210-5214 (2005).

10. Brandt,D.S., Coffman,M.D., Falke,J.J., & Knight,J.D. Hydrophobic contributions to the membrane docking of synaptotagmin 7 C2A domain: mechanistic contrast between isoforms 1 and 7. Biochemistry 51, 7654-7664 (2012).

11. Davis,A.F. et al. Kinetics of synaptotagmin responses to Ca2+ and assembly with the core SNARE complex onto membranes. Neuron. 24, 363-376 (1999).
