# Supplementary material for: The release of inhibition model reproduces kinetics and plasticity of neurotransmitter release in central synapses
Source: Commun Biol. 2023 Oct 27;6:1091. doi: 10.1038/s42003-023-05445-2 (PMC10611806; doi:10.1038/s42003-023-05445-2)
Supplement: Supplementary file 3 — Description of Supplementary Materials [file 42003_2023_5445_MOESM3_ESM.docx]

**Description of Additional Supplementary Files**

**File name:** Norman_et_al_code.zip.

**Description:** Software toolbox for Monte Carlo simulations of synaptic vesicle fusion models in response to arbitrary [Ca2+](t) stimuli described in this manuscript.
