# Supplementary material for: The release of inhibition model reproduces kinetics and plasticity of neurotransmitter release in central synapses
Source: Commun Biol. 2023 Oct 27;6:1091. doi: 10.1038/s42003-023-05445-2 (PMC10611806; doi:10.1038/s42003-023-05445-2)
Supplement: Supplementary file 4 — Custom MatLab code [file 42003_2023_5445_MOESM4_ESM.zip › SytSim/Norman_et_al_code_description.pdf]

Toolbox for Monte Carlo simulations of synaptic vesicle fusion under 'release of inhibition' models in response to arbitrary [Ca2+](t) stimuli.

The modelling framework and constraining of model parameters are described in the manuscript text.
In brief, a synaptic vesicle (SV) model is specified by providing a number of SNAREpins, a synaptotagmin (Syt) clamping model, a SV replenishment model, and a triggering [Ca2+](t) trace. The **run_simulation** function initialises the environment then calls the **simulate_vesicle** function to generate stochastic simulations of the model in response to the [Ca2+](t) trace. The accuracy of Monte Carlo predictions of vesicular fusion probability depends on the number of simulated release events, therefore the **simulate_vesicle** function continues until a target number of releases is achieved (nRelTot), or up to a given time limit (timeCap). The Results structure returned by the **run_simulation** function contains SV fusion and replenishment times aggregated from all the simulations. Additionally, if requested, it may also contain the detailed record of individual SNAREpin states on each vesicle throughout each simulation. This option is disabled by default because of its excessive memory consumption when many simulations are performed. If it is stored, then this collection is converted into an aggregate record of SNAREpin unclamping over time with the **restructure_pin_record** function.

The only required input for the **run_simulation** code is a [Ca2+](t) trace. All parameters must be passed to the **run_simulation** code as name-value pairs with the name specified in the list below. Code was verified in MATLAB version R2020b.

Model parameters with their default values

CaTimeSeries::dbl (Required). N x 2 array with column 1: time in ms; column 2: [Ca2+](t) in µM.

tryParallel::bool (Optional, default: true). Toggle to attempt distribution of simulations to parallel workers on the 'local' profile, or to run serially.

Seed::int/str (Optional, default: 'shuffle'). Seed for random number generator.

timeCap::dbl (Optional, default: 5). Real computational time limit in mins. Simulations will continue until either nRelTot SV releases are achieved or timeCap is exceeded.

nRelTot::int (Optional, default: 100000). Target number of SV releases to record from simulations. Simulations will continue until either nRelTot SV releases are achieved or timeCap is exceeded.

recordPins::bool (Optional, default: false). Toggle to include a record of individual SNAREpin states throughout each simulation. WARNING: The output can be very large if many simulations are performed.

nSNAREs::int (Optional, default: 6). Number of SNAREpins associated with each SV.

P::dbl (Optional, default: 0). Parameter indicating the Syt clamp architecture to be applied to each SNAREpin (identically). Options:
* P = 0 : All tripartite sites are Syt7.
* 0 <= P <= 1 : Tripartite sites are occupied by Syt1 with probability P and Syt7 with probability (1-P).
* P = 1 : All tripartite sites are Syt1.
* P = 2 : No clamp on primary site.
* P = 3 : No clamp on tripartite site.

kon::dbl (Optional, default: 1). Ca2+ binding rate (µM-1 ms-1) to both Syt1 and Syt7. Used to compute koff.

kin::dbl (Optional, default: 100). Membrane insertion rate (ms-1) for both Syt1 and Syt7. Used along with koff to compute kout.

fmodel::str (Optional, default: 'exponential'). Model for the SV fusion rate depending on the number of unclamped SNAREpins. Options:
* 'instant' : SV fuses instantaneously when exactly R SNAREpins are unclamped.
* 'step' : SV fusion rate is 0 when fewer than R SNAREpins are unclamped. When R or more SNAREpins are unclamped the SV fusion rate is a constant (default: 10 ms-1).
* 'exponential' : SV fusion rate is given by the Arrhenius equation (i.e. it grows exponentially in the number of unclamped SNAREpins) as described in the paper.

R::dbl (Optional, default: 3). Flexible parameter to be used as required by each SV fusion model.

rmodel::str (Optional, default: ‘none’). Model for SV replenishment after a fusion event at that site. Options:
* ‘none’ : No SV replenishment after fusion.
* ‘fixed’ : SV replenished after a fixed refractory time (default: 2.5 ms).
* ‘delay’ : SV replenished after a fixed refractory time (default: 2.5 ms) plus a random repriming time drawn from a constant rate (default: 0.02 ms-1).

Description of the algorithm

If a parallel pool named ‘local’ is available then simulations are distributed across all available workers (nWorkers). The **simulate_vesicle** function and its parameters are passed to each worker and simulations continue until each achieves nRelPerWorker releases where nRelPerWorker = ceil(nRelTot / nWorkers). Markov chains are constructed for the specified model with the initial condition that all synaptotagmin clamps are in the Ca2+ unbound state S0. Simulations proceed according to the direct Gillespie algorithm. This ‘next-time’ calculation step of this algorithm involves solving the integral of the [Ca2+](t) trace. This is computed prior to the simulation loop and converted into a piecewise polynomial object which can be interpolated much more quickly than the original integral can be solved numerically. After simulations have completed the results are aggregated from each worker and collected into the Results structure object.

Contents of the Results structure

releaseTimes::dbl Unsorted array of times at which SV fusion events occurred (ms).

repleniTimes::dbl Unsorted array of times at which SVs were replenished following a fusion event (ms).

PinRec::cell(dbl) A record of SNAREpin clamp status for each simulated vesicle, as follows:
* Column 1 : Time (ms).
* Column 2 : Number of SNAREpins with an unclamped primary site.
* Column 3 : Number of SNAREpins with an unclamped tripartite site.
* Column 4 : Number of SNAREpins with both primary and tripartite sites released.
* Note, a new row is added only when a SNAREpin becomes unclamped or a clamp is restored.

metadata::struct Contains model parameters, including a copy of CaTimeSeries, as well as the total number of SVs simulated (nVesicleSites) and the total run time (runTime).

Passing the Results structure through *restructure_pin_record* unpacks the PinRec field and replaces it with the following fields:

priPins::dbl An (nSNAREs+2) x N array in which the first row indicates time (ms) and rows 2 to nSNAREs+2 indicate the total number of simulated SVs which had 0 to nSNAREs unclamped primary sites respectively Note that, due to the initial condition, the first column should have the row 1 element equal to nVesicleSites and all other elements equal to zero.

triPins::dbl As in priPins but for unclamped tripartite sites.

freePins::dbl As in priPins but for when both primary and tripartite site are released.

Pins_on_release::dbl A 1D array with length equal to releaseTimes. Each element indicates how many SNAREpins were unclamped at the instance of fusion for the corresponding element in releaseTimes.

Example

The package includes an example script, **EXAMPLE.m**, which demonstrates how to generate results for a selection of models using the provided functions. This example uses the paired-pulse [Ca2+](t) trace illustrated in Figure 4A of the manuscript (data in *CalciumTraces/2AP_40nm_20ms.csv*) to stimulate the three limiting cases of clamping architecture considered in the paper. These were Syt1P (corresponding to P = 3); Syt1P/Syt1T (P = 1); and Syt1P/Syt7T (P = 0). These three models were simulated both with no SV replenishment (rmodel = ‘none’) and with the replenishment model used for Figures 5 – 6 when considering responses to AP bursts (rmodel = ‘delay’). The outputs of this script are provided in the *Results* directory.
